# Hydrochlorothiazide-associated cutaneous pseudolymphoma

**DOI:** 10.1016/j.jdcr.2025.09.034

**Published:** 2025-10-10

**Authors:** Martha Chavez, Alicia Goldenberg, Drew Kuraitis

**Affiliations:** a Department of Pathology, University at Buffalo, Buffalo, New York; b Department of Pathology, Roswell Park Comprehensive Cancer Center, Buffalo, New York; c Department of Dermatology, Roswell Park Comprehensive Cancer Center, Buffalo, New York

**Keywords:** atypical lymphoid infiltrate, cutaneous lymphoma, cutaneous T-cell lymphoma, hydrochlorothiazide, mycosis fungoides, pseudolymphoma

## Introduction

Cutaneous pseudolymphoma is a benign lymphoid proliferation that clinically and histologically mimics cutaneous lymphoma. While often idiopathic, it is frequently associated with identifiable triggers such as infections, insect bites, and medications, including antihypertensives.^1^^,^^2^ Additionally, the interval between drug exposure and symptom onset varies widely, making identifying the diagnosis even more complex.^1^ Differentiating pseudolymphoma from true lymphoma necessitates clinicopathologic correlation, through medication review, and observation of symptom resolution following drug withdrawal. Although uncommon, recognizing pseudolymphoma is important to avoid unnecessary oncologic treatment. This case highlights a nonphotodistributed, hydrochlorothiazide (HCTZ)-associated pseudolymphoma mimicking mycosis fungoides (MF), the most common subtype of cutaneous T-cell lymphoma (CTCL).

## Case presentation

**Fig 1:**
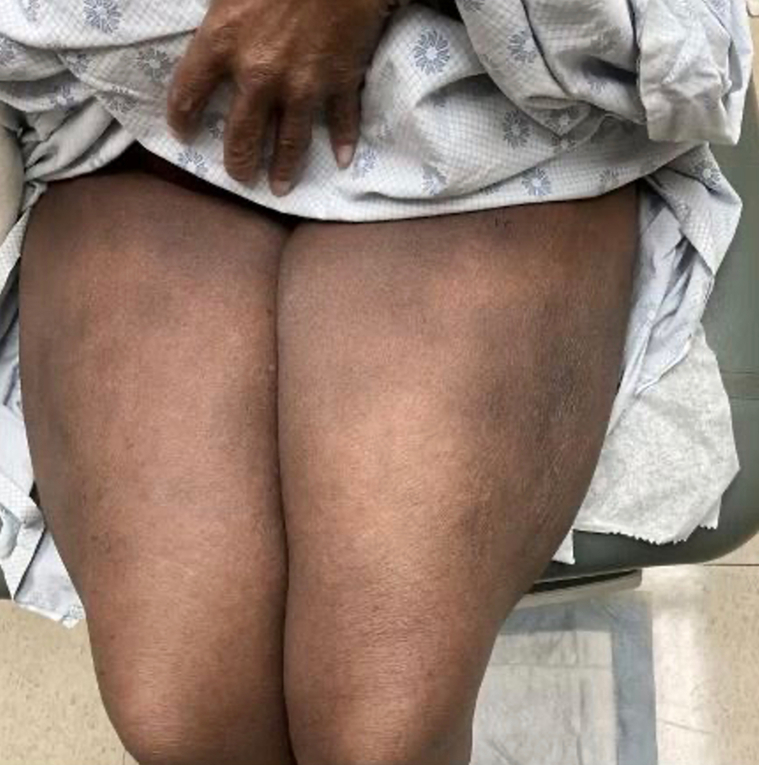
Cutaneous pseudolymphoma. *Rounded* hyperpigmented patches and thin plaques to the thighs at initial presentation.

A 69-year-old Black woman with a medical history of hypertension presented to the dermatology clinic with a rash of approximately 1 month’s duration. Her rash started with itching on the scalp, and later spread to the trunk and extremities. She also reported generalized pruritus that was not restricted to skin lesions. The patient also reported unintentional weight loss over the previous 6 to 7 months. Her medications at the time of presentation included carvedilol, furosemide, and pravastatin. Physical examination revealed hyperpigmented round and ovoid patches and thin scaly plaques, most prominent to the abdomen and thighs (Fig 1), with an estimated body surface area involvement of at least 20%. Lesion distribution favored sun-protected areas. Based on lesion morphology along with reported weight loss, there was a concern for CTCL, particularly mycosis fungoides. Three punch biopsies were performed for histopathologic analysis.

**Fig 2:**
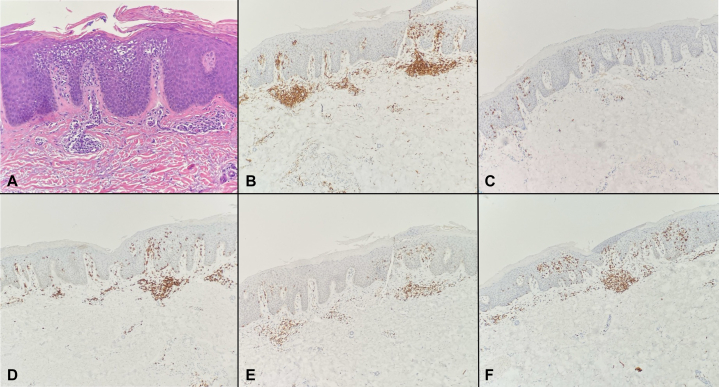
Micrographs from the left forearm stained with hematoxylin and eosin **A,** CD4 **B,** CD8 **C,** CD7 **D,** CD5 **E,** and CD2 **(F)** representing all biopsies due to similar histologic findings. Hyperchromatic lymphocytes are tagging along the dermoepidermal junction with areas demonstrating lymphocyte exocytosis **(A)**. There is a predominance of CD4 positive lymphocytes when compared to CD8 **(B,** and **C)**. CD7 expression appears diminished **(D)**. CD5 and CD2 are preserved **(E,** and **F)**.

Histopathology revealed an atypical lymphoid infiltrate composed of hyperchromatic lymphocytes tagging along the dermoepidermal junction with associated areas of lymphocytic exocytosis, observed across all 3 biopsies (Fig 2, *A*). Immunohistochemistry demonstrated a CD4:CD8 ratio of approximately 6:1 (Fig 2, *B* and *C*) with diminished CD7 expression, and preserved CD2 and CD5 (Fig 2, *D*, *E*, and *F*). A Periodic Acid Schiff stain was negative for fungal organisms. T-cell receptor gene rearrangement studies by polymerase chain reaction did not identify a clonal T-cell population. Although the histologic and immunophenotypic features raised concern for CTCL, the absence of T-cell clonality and the overall findings were insufficient to establish a definitive diagnosis. Therefore, the infiltrate was interpreted as an atypical lymphoid proliferation, falling short of the diagnostic threshold for CTCL at the time of biopsy.

The patient underwent further workup. Complete blood count, comprehensive metabolic panel, and lactate dehydrogenase were within normal limits. A whole-body positron emission tomography scan did not demonstrate evidence of metabolically active lymphoma. Flow cytometry performed on peripheral blood did not demonstrate definite evidence of a lymphoproliferative neoplasm. Upon further review of her medical history and medication use, the patient recalled that she had started HCTZ for leg edema before the onset of her symptoms. Given the timing, absence of systemic or clonal evidence of lymphoma, and histologic features, a diagnosis of HCTZ-induced pseudolymphoma was favored. The patient was advised to avoid further use of HCTZ, and over time, the patient's symptoms resolved, supporting the presumed diagnosis of HCTZ-induced pseudolymphoma. She has been followed for over 1 year since resolution without recurrence of rash or generalized pruritus.

## Discussion

The clinical and histologic overlap between pseudolymphoma and true lymphoma presents a diagnostic challenge. Both conditions may manifest as patches, infiltrated plaques, or papules that are indistinguishable clinically, necessitating histologic evaluation for differentiation.^3^ Lymphomatoid drug reaction (LDR) is a known pseudolymphoma that mimics MF.^4^ Shared histologic features include dermal and epidermal lymphoid infiltrates with epidermotropic CD4-positive predominant T-cell infiltrates with reduced CD7 expression.^4^ In addition, LDR typically shows a polymorphous infiltrate with moderate atypia, eosinophilic spongiosis, and a band-like or perivascular distribution.^4^ The infiltrate usually contains small to medium lymphocytes with moderate atypia and admixed eosinophils.^4^ CD8 positive intraepidermal lymphocytes and only mild reduction of CD7 (>70% loss is unusual) are more suggestive of LDR than MF.^4^ In contrast, MF more often presents with a monomorphous infiltrate of atypical lymphocytes, prominent epidermotropism, marked CD7 loss, and clonal T cell receptor gene rearrangements.^4^ Eosinophils and spongiosis may be characteristic of LDR, but typically absent in MF.^4^ Our patient’s presentation - polymorphous infiltrate, modest CD7 reduction, CD4-predominant dermal infiltrate, negative TCR clonality, and recent HCTZ exposure supported the pseudolymphoma LDR diagnosis over MF.

Associations between HCTZ use and early-stage mycosis fungoides has previously been reported. In 1 study, patients demonstrated remission after stopping HCTZ and also demonstrated recurrence upon rechallenge, raising concern for a pseudolymphomatous process mimicking early mycosis fungoides that was spurred by HCTZ.^5^ Another study investigating if an association exists between HCTZ exposure and subsequent diagnosis for CTCL suggested an inverse association between long-term HCTZ exposure and CTCL development, which challenges assumptions about the drug’s causative role in true CTCL development.^6^ These contradictory findings emphasize the subtleties between drug exposure and cutaneous lymphoid proliferation in determining whether or not an entity is a true lymphoma or a pseudolymphoma. Drug-induced pseudolymphomas can also mimic more advanced CTCL presentations like Sezary syndrome, which may include erythroderma and lymphadenopathy, and circulating atypical CD4 T-cells with dense epidermotropic infiltrates histologically.^7^ Distinguishing between true lymphoma and pseudolymphoma entities requires careful correlation with clinical findings, immunophenotyping, molecular studies, and association with drug exposure in order to manage the eruption and also avoid unnecessary oncologic therapy.

This case emphasizes the importance of considering drug-induced pseudolymphoma in the differential of suspected CTCL. The patient’s generalized pruritus, weight loss, and atypical lymphoid infiltrates initially raised concern for CTCL; however, despite the histologic findings, the absence of T-cell clonality, negative systemic workup, and the resolution of disease following HCTZ withdrawal instead supports a diagnosis of drug-induced pseudolymphoma. This diagnosis should be considered in cases of new-onset lymphoid infiltrates, especially with recent medication changes. Thus, a conservative approach with elimination of suspected agents can be considered if pseudolymphoma is on the differential diagnosis. Prompt identification and discontinuation of the causative agent can lead to complete remission and spare patients from potentially aggressive lymphoma therapies. This case also underscores the need for vigilant medication reconciliation processes.

## Conflicts of interest

None disclosed.
